# DNA damage precedes apoptosis during the regression of the interdigital tissue in vertebrate embryos

**DOI:** 10.1038/srep35478

**Published:** 2016-10-18

**Authors:** Juan A. Montero, Cristina Sanchez-Fernandez, Carlos I. Lorda-Diez, Juan A. Garcia-Porrero, Juan M. Hurle

**Affiliations:** 1 Departamento de Anatomía y Biología Celular and IDIVAL, Universidad de Cantabria, Santander 39011, Spain

## Abstract

DNA damage independent of caspase activation accompanies programmed cell death in different vertebrate embryonic organs. We analyzed the significance of DNA damage during the regression of the interdigital tissue, which sculpts the digits in the embryonic limb. Interdigit remodeling involves oxidative stress, massive apoptosis and cell senescence. Phosphorylation of H2AX mediated by ATM precedes caspase dependent apoptosis and cell senescence during interdigit regression. The association of γH2AX with other downstream DNA repair factors, including MDC1, Rad50 and 53BP1 suggests a defensive response of cells against DNA damage. The relative distribution of cells γH2AX-only positive, TUNEL-only positive, and cells double positive for both markers is consistent with a sequence of degenerative events starting by damage of the DNA. In support of this interpretation, the relative number of γH2AX-only cells increases after caspase inhibition while the relative number of TUNEL-only cells increases after inhibition of ATM. Furthermore, cultured interdigits survived and maintained intense chondrogenic potential, even at advanced stages of degeneration, discarding a previous commitment to die. Our findings support a new biological paradigm considering embryonic cell death secondary to genotoxic stimuli, challenging the idea that considers physiological cell death a cell suicide regulated by an internal death clock that pre-programmes degeneration.

Histological studies in the past century discovered that most morphogenetic events of the developing organ primordia were accompanied by massive cell death within a portion of their tissue components^1^. Researchers at that time emphasized the contradictory nature of such a cell destruction processes in systems, such as the developing embryo, which requires intense and continuous growth. Similar degenerative phenomena were soon observed in tumor tissues that were also undergoing intense growth. Dying cells in those systems shared an uniform morphological appearance, which was termed apoptosis to distinguish that phenomenon from necrotic cell death that occurred secondary to insults and resulted in the rupture of cell integrity^2^. It was proposed that apoptosis represented some kind of cell suicide to facilitate normal embryonic development or to prevent the spread of a localized lesion, allowing for the survival of the organism as a whole. Initial genetic investigations in *C. elegans*, which were later extended to insects and vertebrates, discovered an evolutionary conserved molecular cascade responsible for apoptosis (see review ref. 3). A family of proteases, termed caspases was identified as central players of apoptosis. However, except for the developing nervous system, mice deficient in caspases showed a poor correlation between their phenotypes and the organ morphology expected to result from the inhibition of cell death during the embryonic development^4^. In addition, we now know that caspases perform other functions in addition to cell death, including a role promoting regenerative proliferation (see refs 4 and 5).

A remarkable process of massive embryonic cell death occurs in the interdigital tissue during digit formation in the developing limb of vertebrates, and this process is rudimentary or absent in species with webbed digits (e.g., bat, duck, and some tortoises). The regression of the interdigital tissue involves massive apoptosis, growth arrest, senescence, and degradation of the extracellular matrix^6^,^7^, and genetic, biochemical, and molecular studies have not yet provided a comprehensive explanation for this process of tissue remodeling. Initiator (caspase-2, 8, and 9) and executioner caspases (caspase-3, 6 and −7) in addition to pro-apoptotic factors of the intrinsic (mitochondrial) apoptotic pathway have been implicated in the regression of interdigital tissue^8^. However, syndactyly is observed only in compound mutants affecting more than one pro-apoptotic factor that is implicated in the permeabilization of the mitochondrial membrane; syndactyly is not observed in mice bearing single or compound caspase mutations, or in mice deficient in the caspase-activating adaptor protein, APAF-1 (reviewed by ref. 7). Similarly, while lysosomes also appear to be active players of interdigital cell death^9^,^10^, mice deficient in cathepsins lack the syndactyly phenotype^11^. In addition, the occurrence of an associated cell senescence process in the regression of interdigits is a striking feature that is difficult to explain^12^. These findings have been largely interpreted as evidence for a functional redundancy between the biological mechanisms responsible for tissue degeneration, allowing each to replace the other should one pathway fail^13^.

The occurrence of proliferation arrest, cell senescence, and apoptosis is suggestive of a common upstream triggering factor. It has been hypothesized that DNA damage might be the primary step of cell death within stem cells which prevents the perpetuation of injured genomes^14^. Eucaryotic cells have evolved a complex DNA repair machinery that is quickly activated following DNA strand breaks. The DNA damage response (DDR) is destined to repair DNA but also results in cell cycle arrest, cell senescence, and apoptosis to ensure genomic stability^15^,^16^. The aim of this study was to determine whether DNA damage precedes the degradative routes observed in the remodeling of interdigits termed the Interdigital Necrotic Zones (INZs).

## Methods

We used Rhode Island chicken embryos from 5 to 8.5 days of incubation (id), which is equivalent to stages 25 to 34 HH, and C57BL6 mouse embryos from 12 to 14 days post-coitum (p.c.). The animal care and handling, and all the experimental procedures were in accordance with the guidelines of the European Communities Council and the Spanish legislation and they were approved by the Service of Animal Health and Welfare of the Regional Government of Cantabria (Reference no. PI-10-15). In all cases studies were done in the third interdigit of the avian leg bud, or the fourth interdigit of mouse samples.

### Immunolabeling, TUNEL assay, and confocal microscopy

The expression of DNA repairing factors was studied by the immunolabeling of vibratome sections or in dissociated interdigital cells from chick embryos ranging from 5 to 8 id or mouse embryos between 13 to 14 days p.c. The use of mouse material allowed us to extend our findings in the avian model to mammals, and increased the availability of commercial antibodies. We employed the following antibodies: γH2AX mouse monoclonal (JBW301, Milipore-Upstate) and rabbit polyclonal (A300-272A, NOVUS); Rad50 rabbit polyclonal (07–1781, Millipore); MDCl rabbit polyclonal (ab41951 Abcam); 53BP1 rabbit polyclonal (A300-272A, Bethyl Lab., Inc); phospho-ATM mouse monoclonal (#4526, Cell Signaling); and active caspase 3 (a generous gift of Dr E. de la Rosa). In most cases, samples were used for double-labeling or for the combined detection of apoptosis by TUNEL assay. Counterstaining using fluorescent-phalloidin (Sigma) or DAPI (Vector Laboratories) was also performed.

For tissue sections, the autopods were fixed in 4% PFA, washed in PBS and sectioned at 100 μm using a vibratome.

To obtain dissociated interdigital mesodermal cells, small squares of the third interdigit from limbs fixed in PFA were dissected free and carefully flattened with a coverslide. The slides were then deep frozen over dry ice to adhere the cells to the glass surface. Cells were permeabilized with 0.5% Triton X-100 (60 min) before incubation with the primary antibody, and after several PBS washes, the cells were incubated with the secondary antibody.

### Experimental manipulation of interdigit regression

Treatments to promote or inhibit interdigit regression were made by the local implantation of heparin acrylic (H5263, Sigma), Affi-Gel Blue beads (BioRad), or anion exchange resin beads (AG1X-2, BioRad; 150 μm in diameter) incubated for one hour in the selected reagents (see ref. 17 for details). Observations were based on the analysis of at least 5 experimental limbs for each treatment and stage.

Interdigital cell death was induced by implanting into chick limbs at id 5 or 5.5 heparin beads (Sigma) incubated in 10 μg/ml of BMP2 (Preprotech) or in the BMP2 analogous compound AB204 (a generous gift of Senyon Choe). In these specimens, we analyzed changes in cell death, DDR markers, and cell senescence. To specifically determine the importance of the DDR in the degenerative process induced by BMP-containing beads, we also performed double treatments with a BMP bead and an anion exchange resin bead incubated with inhibitors of kinases responsible for triggering DDR. For this purpose, we employed KU-60019 (UBPbio) to inhibit ATM (Ataxia Telangiectasia Mutated), VE-821 (UBPbio) to inhibit ATR (Ataxia Telangiectasia Rad3 related) and NU7026 (Selleckchem) to inhibit DNA-PKcs (the catalytic subunit of DNA-dependent protein kinase).

The effect of increased oxidative stress was analyzed by the interdigital implantation of air dryed Affi-Gel Blue beads hydrated in H_2_O_2_. After assaying different concentrations, we selected concentrations of 70 or 100 mM H_2_O_2_, which efficiently induced a moderate increase in cell death.

The effects of inhibiting caspases were analyzed by implanting heparin beads incubated with the pan-caspase inhibitor Q-VD-OPh (TNB-1002 TONBO Biosciences). In all cases, the contralateral untreated limb or limbs treated with a PBS-bead were used as controls.

Treatments with empty beads were employed as controls in all experiments. The absence of effects on interdigit regression of these treatments is illustrated in Supplementary Figure 1.

### Cell senescence and neutral red vital staining

Cell senescence was evaluated by detecting β-galactosidase activity at pH 6 in vibratome sections of limb autopods fixed in 4% glutaraldehyde as previous described in ref. 12.

For vital staining, the autopods were dissected free and immersed in 0.01% neutral red diluted in PBS at 37 °C for 15 to 30 min until positive staining in the areas of cell death was obtained.

### Interdigit explants culture

The third interdigit of chick embryos was dissected free from the leg buds at id 5.5 to 7.5 and cultured for 48 or 72 hr in DMEM (GIBCO) with 10% fetal bovine serum (GIBCO), 100 units/ml penicillin and 100 μg/ml streptomycin. Explants were fixed in 5% trichloroacetic acid and stained with alcian blue to detect chondrogenesis. The specimens were then embedded in araldite or paraffin wax. Tissue sections were obtained and stained with toluidine blue or hematoxylin and eosin.

### Cell nucleofection

The morphological evolution of interdigital cells positive for γH2AX was analyzed in cultures of dissociated interdigit mesoderm. For this purpose, cells were transfected with a construct encoding the human *Btg2* gene cloned into the pCMV6-XL5 vector (SC115914, Origene). The efficiency of electroporation was confirmed using qPCR and immunohistochemistry. After two days of culture, cells were fixed in PFA and labeled with anti-γH2AX in combination with rhodamin-phallodin. The samples were examined using a confocal microscope to correlate changes in the distribution of the actin cytoskeleton with the intensity of γH2AX immunolabeling.

### Confocal microscopy

Samples were examined with a laser confocal microscope (LZEISS LSM 510) using a Plan-Neofluar 10×, 20× or Plan-Apochromat 63× objectives, an argon ion laser (488 nm) to excite FITC fluorescence and a HeNe laser (543 nm) to excite Texas Red. Limb specimens were optically sectioned, and we used the LSM 5 Image Examiner software on a Windows NT-Based PC for stack digitalization in the different experiments.

### Statistics

Cells positive for the different markers of cell death and DDR were counted from photographs of histological specimens taken at the same magnification. Each value represents the mean ± SEM of the percentage of cells positive for the different markers obtained from at least five independent samples from each stage or experimental group. Data were analyzed using Student’s t tests or ANOVAs followed by Bonferroni tests for post-hoc comparisons. Statistical significance was set at p < 0.05.

## Results

### γH2AX-postive DNA damage is a precocious event of interdigital cell death

The initial response of most eucaryotic cells to repair DNA damage is the phosphorylation at the break region of substrates, such as the histone variant H2AX that forms γH2AX (H2AXS139PO4; ref. 18) that facilitates the recruitment of additional DDR mediators. Therefore, we analyzed the extent and potential significance of DNA damage in the regressing interdigit by comparing the γH2AX expression pattern with conventional markers of interdigital cell death and tissue regression.

**Figure 1:**
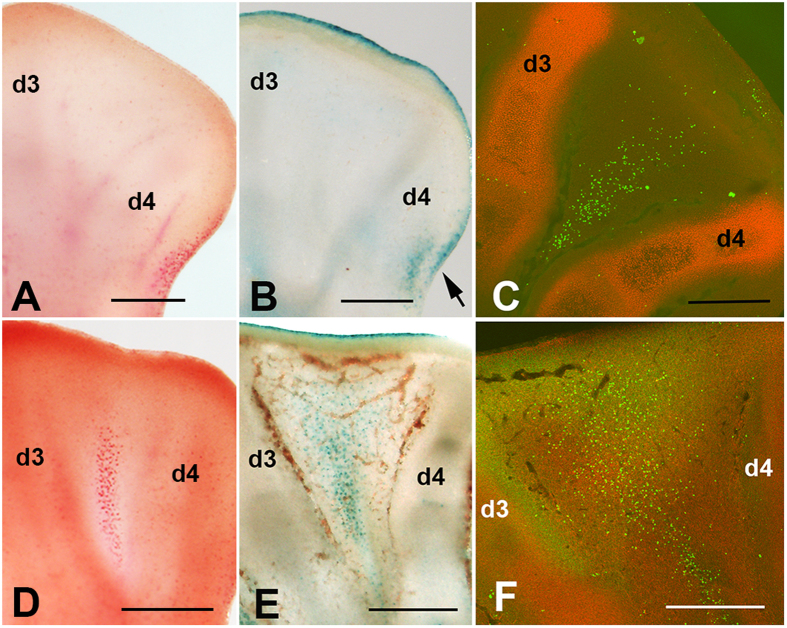
Sequence of interdigit regression in the chick leg bud following neutral red vital staining (**A,D**); β-gal assay for cell senescence (**B,E**); and γH2AX immunolabeling (**C,F**). (**A**–**C**) Show autopods at id 6, indicating that, at this stage, only γH2AX immunolabeling (**C**) shows evidence of initial degeneration. (**D–F**) Show autopods at id 7 and demonstrate that the positivity of the third interdigit for neutral red (**D**) and β-gal labeling (**E**) is relatively less intense than the distribution of γH2AX -positive cells (**F**). Arrow indicate the posterior necrotic zone (PNZ) in (**B**). In all pictures, digit 3 (d3) and digit 4(d4) are indicated to establish the limits of the third interdigit. In (**C**), γH2AX immunolabeling is combined with SOX9 which marks the digit cartilage. Bars = 200 μm.

γH2AX immunolabeling appeared as a precise and precocious marker of INZ detectable beginning at 6 id, a time when neutral red vital staining and β-gal labeling for cell death and senescence remain negative (Fig. 1A–C). In subsequent stages (Fig. 1D–F), γH2AX labeling was largely coincident with both neutral red and β-gal, but with more extensive distribution in the interdigit.

**Figure 2:**
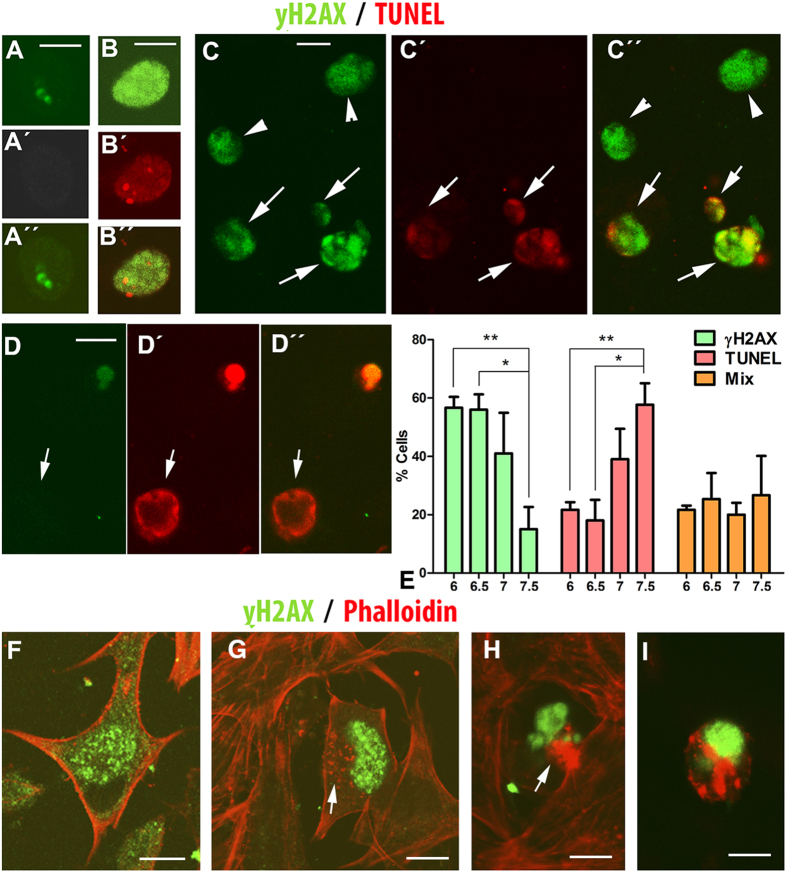
(**A**–**D**) Detailed views of chick interdigital cells, isolated from the third interdigit at id 6.5 and 7, showing different levels of positivity for γH2AX immunolabeling (green) and TUNEL (red). (**A**) Cells at the initial stages of γH2AX positivity areTUNEL-negative. A, shows γH2AX immunolabeling; A´ shows TUNEL labeling; and A´´, shows a merge image. (**B**) γH2AX positive cell at the initial stages of TUNEL positivity. B, shows γH2AX positive cell; B´ shows TUNEL positivity; and B´´ shows the merge image. (**C**) A small group of interdigital γH2AX-positive cells that include cells H2AX-only-positive cells (arrow heads) and cells double-positive for γH2AX and TUNEL (arrows). C´, shows TUNEL labeling; and C´´ shows the merged image. D–D´´, γH2AX negative and TUNEL positive cell at id 7 (arrow). (**D**) Shows γH2AX labeling; D´ shows TUNEL labeling; and D´´ shows the merged image. (**E**) Graphic representation of the percentage of cells that were γH2AX-only-positive (green columns); TUNEL-only-positive (red); and γH2AX-TUNEL double-positive (orange) in the third interdigit of chick limbs at id, 6, 6.5, 7, and 7.5. **p < 0.01; *p < 0.5. (**F–I**) Interdigital mesodermal cells transfected with hBtg2 gene were cultured for 48 hr and labeled with γH2AX (green) and phalloidin (red) to illustrate the sequence of cell degeneration by correlating the intensity of γH2AX immunolabeling and the disintegration of the actin cytoskeleton. (**F**) Shows the conserved stellated morphology of a cell with mild γH2AX immunolabeling. (**G**) Shows a more advanced level of γH2AX labeling that correlates with a loss of cytoplasmic prolongations and a moderate accumulation of actin filaments in the cytoplasm (arrow). (**H**) Shows a cell with very advanced γH2AX positvity which correlates with massive accumulation of actin in a large cytoplasmic clump (arrow). (**I**) Shows a degenerating cell in the course of detaching from the culture. The cell shape is now rounded and the intense γH2AX labeling correlates with an increased deposition of degenerated actin microfilaments. Bars = 5 μm.

The combined detection of TUNEL and γH2AX revealed three types of degenerating cells in the regressing interdigits: i) cells positive for γH2AX only; ii) cells TUNEL-positive only; and, iii) cells double-positive for γH2AX and TUNEL (Fig. 2A–D). The percentage of cells that were γH2AX-only and TUNEL-only-positive changed over the course of interdigit regression. As shown in Fig. 2E, we found significant differences between the numbers of each type of degenerating cell over the course of interdigit remodeling. At id 6 and 6.5, γH2AX-only-positive cells were predominant (57% and 56%, respectively) and TUNEL-only positive were scarce (21% and 18%, respectively). At id 7, the numbers of γH2AX-only- and TUNEL-only-positive cells were similar. In contrast, at id 7.5, TUNEL-only-positive cells were predominant (58%), while γH2AX-only-positive cells were very scarce (15%). The relative intensity of these two markers in the degenerating cells was also suggestive of a sequence of events. Hence, punctate γH2AX labeling of DNA damage was observed mostly during early stages of interdigit remodeling. In the course of remodeling diffuse pan-nuclear labeling were predominant. These cells were positive for TUNEL in the rate mentioned above. Remarkably, γH2AX labeling in these double positive cells, becomes very intense and compacted, paralleling the pycnotic appearance of TUNEL staining (Fig. 2A–D).

Together, these findings are consistent with a sequence of cell degeneration initiated by DNA damage that involves DNA repair through γH2AX, followed by apoptotic DNA fragmentation and/or cell senescence. In fact, the pattern of pan-nuclear γH2AX labeling is characteristic of cells experiencing replication stress, a feature which precedes cell senescence. Based on this interpretation, TUNEL-only-positive cells might represent a cell population in which the intensity of initial DNA damage, caused by direct DNA breakage or associated with replication stress, precludes the DNA repair response.

To discard that DNA damage was a singular feature of INZ, we analyzed programmed cell death in other areas of the limb (the so-called posterior necrotic zone, PNZ, and the thickened ectoderm of the distal margin of the limb, termed Apical Ectodermal Ridge, AER), and also in other developing organs, such as the heart, where cell death occurs both in mesenchymal cells of the developing septa and valves and also in the muscle of the outflow tract^19^,^20^. Supplementary Figure 2, illustrates the presence of both TUNEL-positive, senescent and γH2AX-positive cells, indicating that the coexistence of these three types of degenerating cells is a general feature of embryonic cell death regardless the type of tissue (epithelium, mesenchyme or muscle).

### Sequence of cell degeneration

To further confirm that DNA damage is an initial step in the degenerative process responsible for interdigit removal, we monitored alterations in the actin cytoskeleton within limb mesodermal cultures transfected with *Btg2*. We have previously shown that the actin cytoskeleton is a precise marker of cell degeneration^21^. In addition, BTG2 is a tumor suppressor factor expressed in the interdigits, and its forced expression in limb mesodermal progenitors induces the accumulation of reactive oxygen species (ROS), cell death, and senescence^12^. As expected, the number of cells positive for γH2AX increased significantly in cultures overexpressing *Btg2* (Supplementary Figure 3). Although, our observations would require further comprobation by living imaging procedures, a detailed examination of the actin cytoskeleton allowed us to establish a temptative sequence of degeneration based on the intensity of γH2AX positivity and the level of disintegration of the actin cytoskeleton (Fig. 2F–I). Cells with moderate levels of γH2AX-positive foci maintained their characteristic stellated appearance with a peripheral distribution of actin bundles (Fig. 2F). More intense levels of γH2AX positivity were associated with a reduction or loss of cell prolongations that were accompanied by occasional deposition of fine clumps of actin-positive material (Fig. 2G). Intensification and predominant pan-nuclear distribution of γH2AX labeling was accompanied by cell rounding, loss of peripheral actin fibers, and dramatic intracytoplasmatic precipitation of actin-positive material (Fig. 2H). In the final stages of degeneration, the nuclei appear pyknotic, and cells lost contact with their neighbours being detached into the culture medium (Fig. 2I).

### The activation of the DNA damage repair response in interdigital cells undergoing cell death

**Figure 3:**
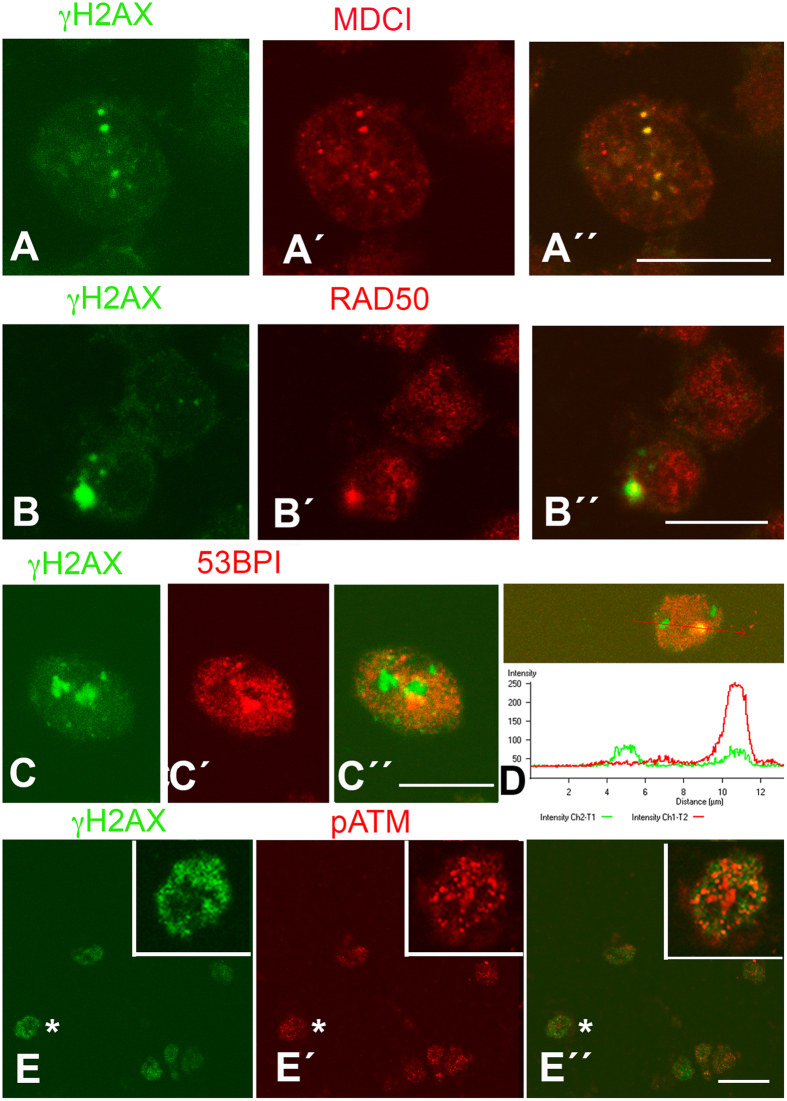
Mouse interdigital cells at day 13.5 pc immunolabeled for γH2AX (**A–C**) in combination with MDC1 (A´), RAD50 (B´) and 53BP1 (C´) to show the activation of DDR mediators prior to overt cell degeneration. A´´, B´´, and C´´ are merged images. (**D**) Pixel intensity plot of the region indicated by red arrow showing the labeling distribution of 53BP1 (red) and γH2AX (green) in an interdigital cell similar to that shown in (**C**) Note the presence of isolated γH2AX foci and a region of colocalization with 53BP1. (**E**) Chick interdigital cells at id 6 immunolabeled for γH2AX (**E**) and pATM (E´). E´´ is the merged image. Inset is a detailed view of the cell indicated by asterisk. Bars in (**A–C)** = 10 μm; bar in (**E**) = 30 μm.

The data described above suggest that precocious DNA damage precedes apoptosis in regressing interdigits. Apoptotic DNA breaks might trigger the initial phosphorylation stages of DDR; however, several DDR mediators are cleaved by caspases, which precludes their activation in apoptotic cell death^22^. Therefore, we asked whether the phosphorylation of H2AX is accompanied by the activation of other DNA repair factors. To investigate this, we analyzed by immunohistochemistry the presence of a panel of DNA repair factors in the interdigit mesoderm over the course of remodeling (Fig. 3).

γH2AX-positive cells were also labeled with anti-phospho-ATM (Fig. 3E). Positivity was also observed for the mediator of DNA damage checkpoint protein 1 (MDC1) (Fig. 3A). MDC1 is a target of caspases^22^ and it is an important DNA repair factor that regulates cell cycle checkpoints and recruits repair proteins to the DNA. In addition, Rad50 (Fig. 3B), which marks the initiation of DNA repair, and 53BP1 (Fig. 3C), which is a chromatin-binding factor downstream of γH2AX and MDC1 in the DDR cascade, were also activated in the interdigital cells that were positive for γH2AX. Consistent with the occurrence of a sequence of degenerative events, the mentioned factors often co-localize with γH2AX (Fig. 3) at the beginning of the degenerative process, but co-localization foci are scarce at advanced stages of interdigit remodeling.

Together, these observations support the hypothesis that interdigit remodeling is initiated by non-apoptotic DNA damage.

### Activation of caspases and γH2AX labeling

**Figure 4:**
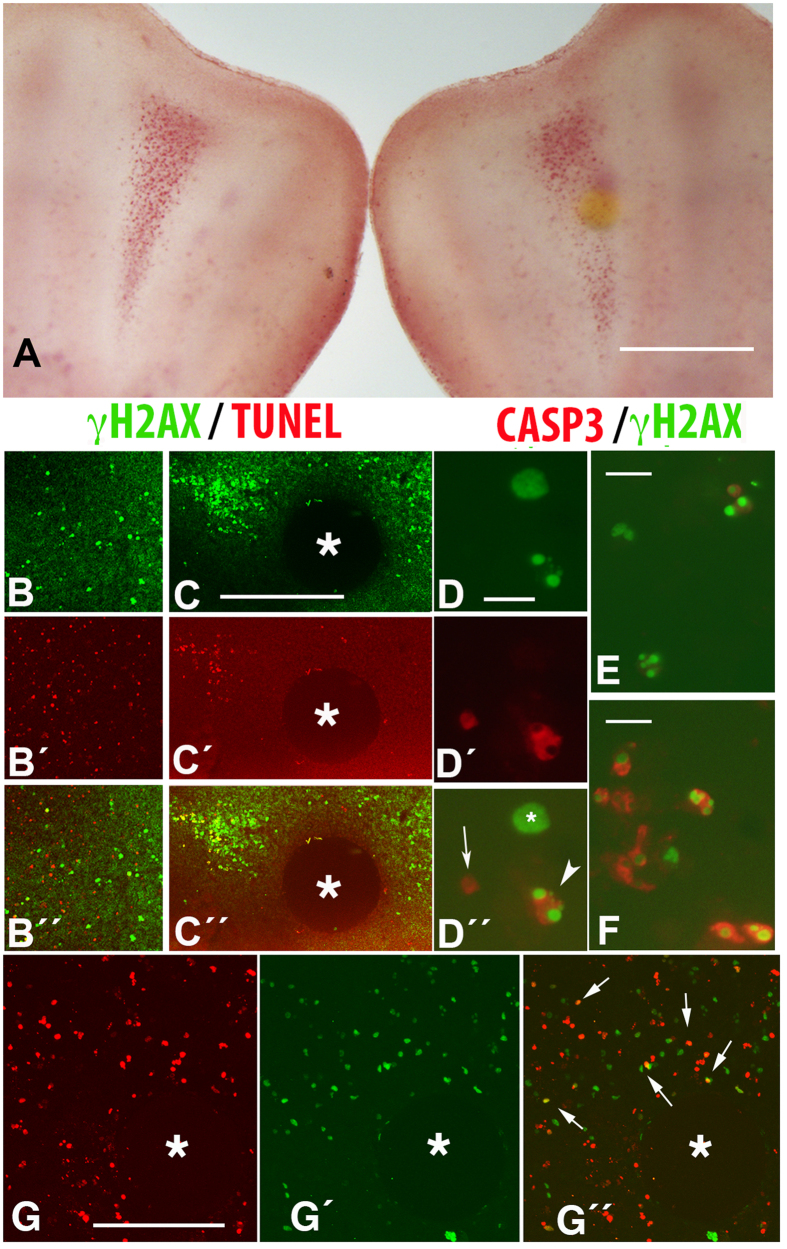
(**A**) Control (left) and experimental (right) autopods 24 hr after the interdigital implantation of a bead incubated with the pan-caspase inhibitor and following vital staining with neutral red. Note the moderate diminution of cell death around the bead. Bar = 200 μm. (**B,C**) Are confocal images of control (**B**) and experimental (**C**) interdigits 24 hr after the interdigital implantation of a bead (*) bearing the pan-caspase inhibitor. Note the reduction in TUNEL-positive cells (C´; red labeling) in comparison with the contralateral, control interdigit (B´). Note also the major abundance of cells γH2AX-positive in the experimental interdigit (**C**, green labeling) in comparison with the control (**B**). B´´ and C´´ are the merged images of **B**–B´ and **C**–C´. Bar for (**B,C**) = 100 μm. **D**–D´´ Squash of the interdigital mesoderm at id 7.5 after immunolabeling with anti-γH2AX (green in **D** and D´´) and active caspase 3 (red in D´and D´´), to show that cells positive for γH2AX can be either negative (* in D´´) or positive (arrowhead in D´´) for caspase 3. Arrow shows a cell positive for active caspase 3 but negative for γH2AX. Bar = 15 μm. (**E,F**) Squashes of the third interdigit at stage 6.5 (**E**) and 7.5 (**F**) to show the increase in active caspase immunolabeling in the course of interdigit regression, Bar = 10 μm. **G**–G´´ are confocal pictures of the interdigital mesoderm showing TUNEL (**G**, red) and γH2AX (G´, green) labeling 10 hr after the implantation at id 5.5 of a H_2_O_2_ bead (*). G´´ is the merged image. Note the presence of cells positive for γH2AX only (green), cells TUNEL-positive only (red), cells double-positive for γH2AX and TUNEL (arrows in G´´). Bar = 100 μm.

The involvement of caspases in the degenerative process was explored by interdigital implantation of the pan-caspase inhibitor Q-VD-OPh. Beads containing Q-VD-OPh and implanted in the interdigits did not cause syndactyly, but they moderately inhibited the number of dying cells as detected by neutral red staining (Fig. 4A). Immunohistochemical analysis of these treated interdigits revealed that the number of TUNEL-positive, but not the number of γH2AX-positive cells, was reduced in the area around the Q-VD-OPh-bead (Fig. 4B,C). Consistent with this finding, double immunolabeling for active caspase 3 and γH2AX revealed the presence of three types of degenerating cells in the regressing interdigits: cells positive for γH2AX but negative for active caspase 3; cells positive for active caspase 3 and negative for γH2AX; and cells double-positive for caspase3 and γH2AX (Fig. 4D). The number of cells positive for active caspase3 immunolabeling was more abundant at advanced stages of interdigit regression, and the intensity of labeling within each cell was also higher (Fig. 4E,F). In addition, cells double positive for active caspase 3 and γH2AX exhibited signs of advanced degeneration, such as nuclear fragmentation. As expected, TUNEL-positive cells were positive for active caspase 3 as previously reported (not shown).

### Oxidative stress and γH2AX labeling

It is well known that interdigital tissue regression is accompanied by increased oxidative stress^23^,^24^. Taking into account that oxidative stress is a major factor responsible for DNA damage, we investigated whether local increase in oxidative stress induced a pattern of cell degeneration similar to that observed during physiological regression. To this end, at id 5 or 5.5 we implanted in the prospective interdigit mesoderm heparin beads incubated in H_2_O_2_ (70 or 100 mM). Ten hours after bead implantation, a number of cells undergoing degeneration were observed around the bead. As shown in Fig. 4G, these cells showed a pattern of γH2AX and TUNEL labeling similar to that found in the physiological regressing interdigits, including the presence of γH2AX-only, TUNEL-only and TUNEL-γH2AX double-positive cells (Fig. 4G–G´´).

### The chondrogenic potential of interdigital cells is fully conserved until the establishment of overt DNA damage and apoptotic degeneration

**Figure 5:**
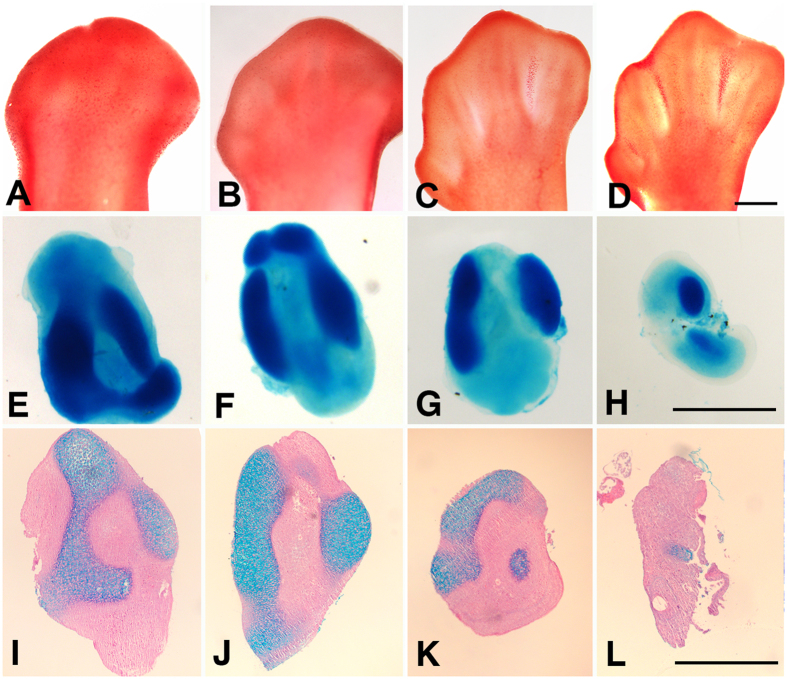
(**A–D**) Embryonic chick autopods at id 5.5 (**A**), 6 (**B**), 7 (**C**) and 7.5 (**D**) vital stained with neutral red to illustrate the sequence of interdigital cell death in the regressing interdigits. Bar = 300 μm. (**E–H**) Interdigit explants from embryonic limbs at id 5.5 (**E**), 6 (**F**), 7 (**G**) and 7.5 (**H**) stained with alcian blue after 72 hr of culture to correlate the chondrogenic outcome relative to the stage of interdigital cell death shown in (**A**–**D**) Bar = 200 μm. (**I–L**) Tissue sections of interdigit explants of limbs at id 5.5 (**I**), 6 (**J**), 7 (**K**), and 7.5 (**L**) cultured for 72 hr. The explants were first whole-mount stained with alcian blue and next paraffin wax embedded and sections stained with hematoxylin and eosin. Note the presence of alcian blue stained cartilage nodules in all samples. Bar = 200 μm.

It is currently believed that cell death in embryonic systems is irreversibly and temporally programmed in the zones of prospective degeneration prior to the appearance of dying cells. The term “internal death clock” has been proposed to describe this phenomenon^25^. In previous studies, we have shown that interdigital cells prior to cell death are fully competent to differentiate into cartilage and, *in vivo*, are able to form an extra-digit^26^. Therefore, we looked to determine the stage at which the interdigit mesoderm loses chondrogenic potential relative to the appearance of DNA damage and massive apoptosis. To this end, we dissected the third interdigit of leg autopods at different stages of development and cultured the explants. As shown in Fig. 5, 100% of the interdigits at id 5.5 (n = 10) and 6 (n = 10) developed one or various cartilages. By id 7, the interdigits formed small cartilages, but this differentiation was still observed in 100% of the samples (n = 10). By id 7.5, the formation of cartilage was observed in 4 out of 10 cultured interdigits, and the size of the cartilage was significantly reduced in comparison to previous stages. These data argue against the occurrence of an internal death clock that is present in advance of the onset of degeneration.

### Experimental induction of interdigital cell death replicates the degenerative sequence observed during physiological interdigit regression

**Figure 6:**
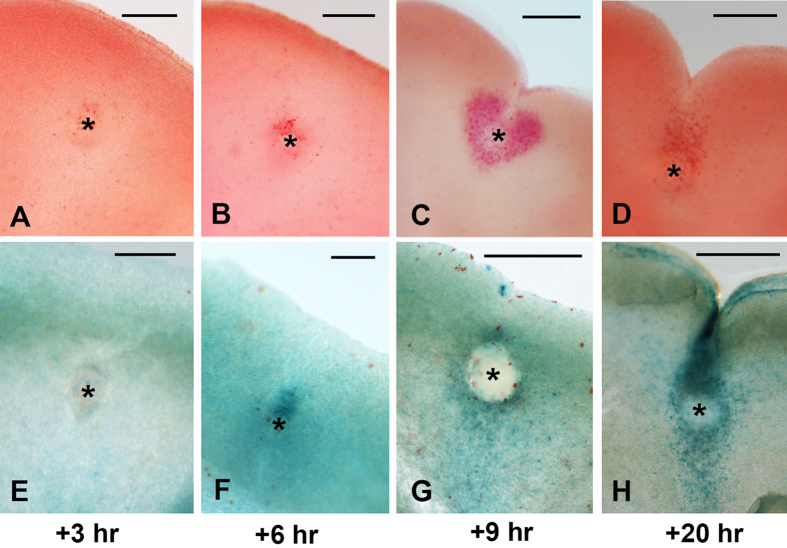
(**A–D**) Interdigital spaces showing the appearance of neutral red-positive labeling (**A–D**) and β-gal activity (**E–H**) at 3 (**A,E**), 6 (**B,F**), 9 (**C,G**) and 20 hr (**D,H**) after the implantation of a BMP-bead (*) at id 5.5. Note that positive labeling is first identified 6 hr after bead implantation, but an intense area of tissue regression is not observed until 9 hr after the implantation of a BMP bead. Bars = 200 μm.

**Figure 7:**
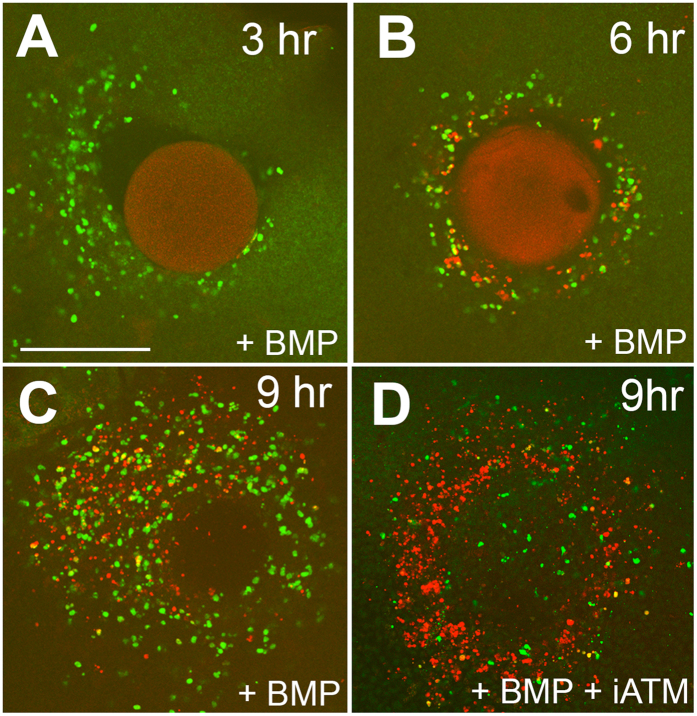
Distribution of γH2AX (green)- and TUNEL (red)-positive cells in the interdigital mesoderm 3 (**A**), 6 (**B**) and 9 (**C**) hr after the implantation of a BMP bead. Note the precocious appearance of γH2AX-positive cells 3 hr after bead implantation and the progressive increase of TUNEL-positive cells 6 and 9 hr after bead implantation. (**D**) Shows an interdigit 9 hr after the implantation of a BMP bead in combination with a bead incubated in Ku-60019 to inhibit ATM indicating a relative increase in the number of TUNEL-positive cells and a decrease in the number of γH2AX-positive cells (compare with (**C**)). Bar = 140 μm.

Mouse genetic and chick microsurgical experimental studies have shown that the interdigital activation of BMPs induces the precocious degeneration of the AER riming the interdigit and massive apoptosis in the interdigital mesoderm^27^,^28^. In addition, the interdigital application of microbeads containing BMPs is followed by the precocious degeneration of the interdigital mesoderm, reproducing the physiologic regression mechanisms^17^. Labeling for neutral red and β-gal was almost unidentifiable at 3 and 6 hr after bead implantation (Fig. 6A,B,E,F). Beginning at 9 hr after bead implantation, both neutral red (Fig. 6C) and β-gal (Fig. 6G) labeling showed clear evidence of cell degeneration and senescence, respectively, which was increased in subsequent stages (Fig. 6D,H). In contrast, γH2AX immunolabeling identified a zone of intense labeling in the mesoderm around the bead as early as 3 hr after bead implantation (Fig. 7A). At this short period, most of the degenerating cells were γH2AX positive, and only few were TUNEL-positive. By 6 hr after bead implantation, the numbers of TUNEL- and γH2AX-positive cells were balanced, and at later periods, TUNEL-positive cells were predominant (Fig. 7B,C).

Double treatment with BMP-beads and beads incubated with specific inhibitors of kinases implicated in DDR (ATM, ATR, or DNA-PKcs) delayed the appearance of γH2AX expression within cells. The strongest inhibition was observed following co-treatment with Ku-60019, which selectively inhibits ATM (Fig. 7D). The weakest inhibition was obtained with co-treatments with inhibitors of ATR inhibitors.

## Discussion

The cellular basis of the remodeling processes that occurs during embryonic development has been the focus of intense research over the decades, yet the mechanisms remain poorly understood. The elimination of the interdigital tissue to sculpt the digits in the embryonic limb of vertebrates is a popular model for studying embryonic programmed cell death. At the cellular level, interdigit regression includes massive apoptosis^29^, cell senescence^12^, and lysosomal-mediated cell death^9^,^10^. Our present findings demonstrate that prior to caspase activation, cells destined to die showed the activation of a DDR that involves the phosphorylation of histone H2AX and the nuclear recruitment of various DDR factors. Furthermore, the identification of γH2AX-positive foci in other areas that undergo embryonic cell death, along with the absence of cell death phenotypes (except in the central nervous system) in mice deficient in caspases (reviewed by ref. 7), suggest that precocious DNA damage might be a general feature of embryonic programmed cell death. It is well known that DNA damage in adult and tumor tissues is followed by cell cycle arrest, cell senescence, and apoptosis^16^,^18^,^30^. Based on these findings and considering the sequence of degeneration observed in this study, the damage to the DNA would be the primary event during interdigit regression, while apoptosis and senescence would occur downstream. Furthermore, the activation of the DNA repair mechanism in the interdigital mesoderm suggests that the interdigit regression process is more similar to an injury response resulting from genotoxic stress secondary to changes in local regulatory signals, rather than the traditional view of cellular “suicide” that ensures the outcome of a histogenetic or morphogenetic process in the embryo. Consistent with the hypothesis of an exogenous origin of the cell death signals, we observed that cultured interdigital explants differentiated into cartilage rather than dying even at advanced stages of degeneration. As mentioned above, this finding argues against the presence of an internal death clock that preprograms the initiation of the so-called “programmed cell death”^25^.

The presence of DDR markers along with apoptosis has been reported in embryonic and postnatal retinas and has been interpreted as either protecting^31^ or promoting^32^ the mechanism of tissue regression. Considering that the interdigital mesoderm is positive for ATM immunolabeling, the induction of caspase-positive apoptosis after the local inhibition of ATM indicates that DNA damage is a primary event during interdigit regression. ATM is activated by double-strand DNA breaks and phosphorylates H2AX at S139 forming γH2AX that, in turn, changes the configuration of the chromatin and allows for the recruitment of different DNA repair proteins. TUNEL-positive cells that are negative for markers of DNA damage might represent cells that are induced to undergo cell death through the primary activation of caspases, most likely because the intensity of DNA damage precludes a defense response of DNA repair. It has been shown that cells during the course of DNA repair deliver paracrine factors that trigger apoptosis and promote cell senescence in the neighboring cells^30^. In addition, the signals generated during DDR target the mitocondria promoting apoptosis^33^. Hence, the temporal sequence in the appearance of TUNEL- and γH2AX-positive cells and their relative numbers over the course of spontaneous or experimentally induced interdigital cell death strongly support our interpretation. Furthermore, we also observed that the number of TUNEL-positive, but not γH2AX-positive cells, are reduced after treatments with a pan-caspase inhibitor. Our findings explain why interdigital tissue regression is delayed but not abrogated in mice deficient in the caspase activating factor APAF1^13^.

Although the identification of the cause of DNA damage was not a primary aim of this study, two potential factors could function in the system. Oxidative stress increases in the interdigits in the course of remodeling^12^,^24^,^34^. ROS are major factors responsible for DNA damage and replicative stress in a variety of tumor and tissue types, and we have shown here that local application of H_2_O_2_ induces a pattern of degeneration similar to that occurring under physyiological interdigit regression. A second, non-exclusive possibility is that DNA breaks are associated with changes in chromatin configuration induced by high motility transcription factors (HMG). These transcription factors compete with histone H1 to modify the functional structure of chromatin, and this function might involve the tight regulation of DNA breaks and repair mechanisms to ensure the integrity of the genome^35^,^36^. In the regressing interdigits, various HGM genes including Hmgn1 and Sox9 are expressed (refs 37,38 and personal observations). *Sox9* in combination with HMGN1^37^ and Tgfβ signaling^39^ plays a central role in determining whether skeletal progenitors differentiate into chondrocytes or remain undifferentiated to be removed via induced cell death. High levels of Sox9 lead to chondrogenesis and the absence of Sox9 results in massive cell death^40^. Furthermore, the interdigital application of Tgfβ induces the intense and precocious (30 min) up-regulation of *Sox9*, which prevents cells from dying and instead induces cartilage differentiation^39^.

In summary, our study proposes a new biological paradigm for embryonic cell death. Unlike previous interpretations of the programmed fate of specific tissue components of the embryo, our findings indicate that cells are removed because they receive noxious stimuli. Based on our results, cell death is not a positive cell behavior but rather cells try to survive to local stress.

## Additional Information

**How to cite this article**: Montero, J. A. *et al*. DNA damage precedes apoptosis during the regression of the interdigital tissue in vertebrate embryos. *Sci. Rep.* **6**, 35478; doi: 10.1038/srep35478 (2016).

## Supplementary Material

Supplementary Information
