# Supplementary material for: DNA damage precedes apoptosis during the regression of the interdigital tissue in vertebrate embryos
Source: Sci Rep. 2016 Oct 18;6:35478. doi: 10.1038/srep35478 (PMC5067507; doi:10.1038/srep35478)
Supplement: Supplementary Information [file srep35478-s1.pdf]

DNA damage precedes apoptosis during the regression of the interdigital tissue in vertebrate embryos

Juan A. Montero1, Cristina Sanchez-Fernandez1; Carlos I. Lorda-Diez, Juan A Garcia-Porrero, and Juan M. Hurle*

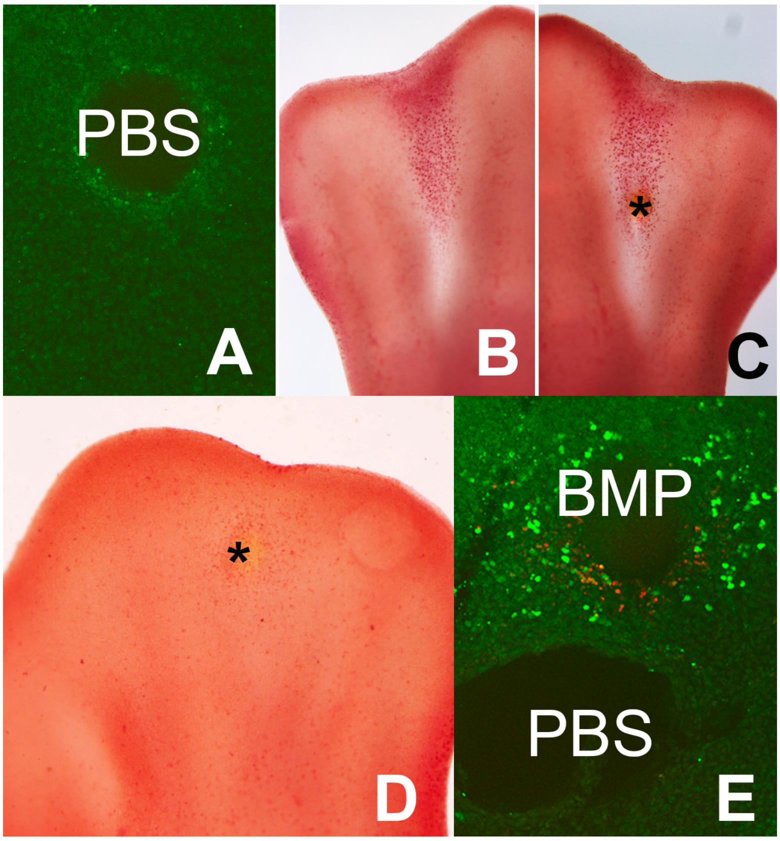

Supplementary figure 1

Interdigital implantation of control beads does not modify the pattern of interdigit regression. A, shows the absence of induced cell death 6 hr after interdigital implantation of a PBS control bead (compare with E). B and C, are neutral red stained interdigits showing that implantation of an acrylic bead (asterisk in C) does not inhibit the normal pattern of cell death in comparison with the contralateral untreated interdigit (B). D, interdigital implantation of a bead incubated in Ku-60019 to inhibit ATM (asterisk) does not induce interdigital cell death. E, TUNEL (red) and γH2AX immunolabeling (green) 6 hr after the implantation of a BMP-bead in combination with a PBS-bead at id 5.5. Note that implantation of a bead incubated in PBS does not change the degenerative effects induced by the BMP-bead. Compare this picture with Figure 7D.

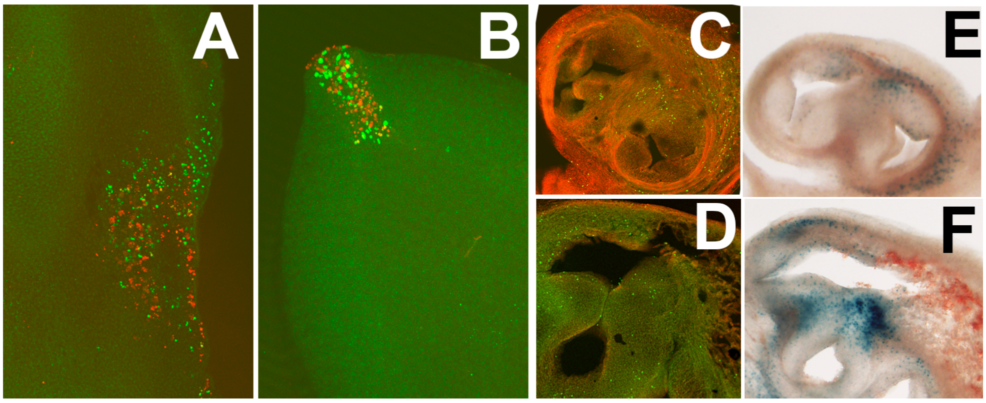

Supplementary figure 2.
All of the analyzed areas of embryonic cell death show cells positive for non-apoptotic DNA damage as detected by γH2AX immunolabeling (green) together with TUNEL-positive (red) apoptotic cells. A and B are gross vibratome sections of the avian limb bud showing the so-called posterior necrotic area (PNZ) at id 6 (A), and the apical ectodermal ridge (AER) at id 4.5 (B). C and D are vibratome sections of the heart at id 7 at the level of the semilunar valves (C) and outflow tract (D), showing positivity for γH2AX immunolabeling. The sections are counterstained with phalloidin (red) to identify actin filaments. E and F are sections similar to C and D, showing senescence-associated β-gal activity.

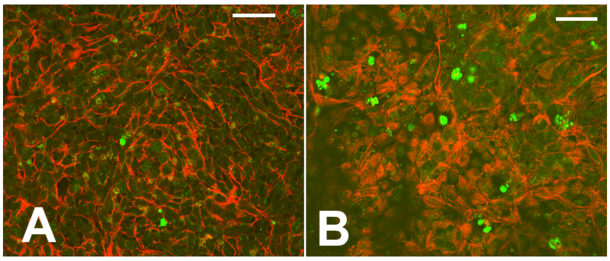

Supplementary figure 3.
Low magnification views of cultures from control limb mesodermal cells (A) and mesodermal cells overexpressing the Btg2 gene (B). Note the abundance of cells positive for γH2AX immunolabeling (green) in cultures transfected with the Btg2 gene. The cultures are counterstained with phalloidin (red) to identify actin filaments Bar = 20 µm
